# Few-shot concealed object detection in sub-THz security images using improved pseudo-annotations

**DOI:** 10.1038/s41598-024-53045-9

**Published:** 2024-02-07

**Authors:** Ran Cheng, Stepan Lucyszyn

**Affiliations:** Department of Electrical and Electronic Engineering, Imperial College London, London, SW7 2AZ UK

**Keywords:** Electrical and electronic engineering, Computational science, Information technology, Scientific data

## Abstract

In this research, we explore the few-shot object detection application for identifying concealed objects in sub-terahertz security images, using fine-tuning based frameworks. To adapt these machine learning frameworks for the (sub-)terahertz domain, we propose an innovative pseudo-annotation method to augment the object detector by sourcing high-quality training samples from unlabeled images. This approach employs multiple one-class detectors coupled with a fine-grained classifier, trained on supporting thermal-infrared images, to prevent overfitting. Consequently, our approach enhances the model’s ability to detect challenging objects (e.g., 3D-printed guns and ceramic knives) when few-shot training examples are available, especially in the real-world scenario where images of concealed dangerous items are scarce.

## Introduction

Recent advancements in convolutional neural networks (CNNs) have positioned deep learning models^1–5^ as leading solutions for object detection in red–green–blue (RGB) image datasets like visual object classes (VOC)^6^, common objects in context (COCO)^7^ and ImageNet^8^. In a very recently published Scientific Reports article, a deep learning model has been successfully applied for detecting concealed objects in high-contrast passive sub-terahertz (sub-THz) imaging^9^. For the purposes of our work, ‘sub-THz’ relates here to the upper-millimeter-wave frequency range from 100 to 300 GHz; the images used in our work are from published measurements undertaken at 140 GHz. In practice, training these deep learning models generally requires large volumes of image data^9–12^, presenting a challenge with expensive sub-THz imaging^13^. In contrast to vision-based methods, the availability of sub-THz imaging datasets is limited, with data collection and annotation more time-consuming. For this reason, when compared to RGB images, there is a scarcity of sub-THz domain image data that is available for training an effective deep learning model. Furthermore, in real-world scenarios, concealed dangerous items (e.g., guns, knives, explosives, etc.) appear infrequently. This rarity leads to a class imbalance in the training dataset, making it more challenging to train an effective deep learning model for detecting such items in (sub-)THz (i.e., at upper-millimeter-wave and sub-millimeter-wave frequencies from 0.1 to 3 THz) security images.

To address the two major challenges of limited dataset and class imbalance, few-shot object detection (FSOD) has recently re-emerged as a promising solution, as it is specially designed for scenarios with limited labeled data of target categories (e.g., guns, knives, explosives, etc.). With a typical FSOD training paradigm^14^, images of objects are divided into two groups: ‘base classes’, which have many examples; and ‘novel classes’, which have limited few-shot examples (i.e., limited number of images used for training). The deep learning model is initially trained on the ‘base classes’ data, followed by fine-tuning using the few-shot ‘novel classes’ data. The two-stage fine-tuning approach (TFA)^15^ stands out as a notable two-stage fine-tuning framework. The region-based CNN (RCNN) series^1–3^ is acknowledged as a benchmark algorithm for two-stage object detection. By freezing all modules within the algorithm, with the exception of the classification layer during the fine-tuning stage, TFA enhances the performance of the Faster-RCNN^3^ in detecting ‘novel classes’ objects, while maintaining its efficiency in detecting ‘base classes’ objects. By employing the fine-tuned detector, trained using the TFA framework, label-verify-correct (LVC)^16^ incorporates a vision transformer (ViT)^17^. This approach extracts high-quality pseudo-annotations from additional unlabeled datasets, significantly improving the model’s ability to detect ‘novel classes’ objects. It is worth noting that certain FSOD frameworks^18–21^ substitute base-training with self-supervised pre-training on extensive datasets, while other machine learning frameworks (e.g., detection with transformers using region priors (DETReg)^22^) eliminate the fine-tuning step by leveraging support images to condition the model. However, in the (sub-)THz domain, where training examples are exceedingly scarcer, when compared to RGB images, fine-tuning methods like TFA and LVC are particularly well-suited for (sub-)THz imaging applications.

FSOD is primarily utilized in customized applications where certain categories of data or examples are limited. In the realm of computer vision research on FSOD, it is common for researchers to randomly select 60 object classes as ‘base classes’ and the remaining 20 classes as ‘novel classes’ within the COCO RGB images dataset. However, due to the highly customized nature of FSOD, there are no explicit open-source examples of applying FSOD. Notably, our work is among the first to apply FSOD in the sub-THz imaging, where FSOD is especially well-suited for detecting rarely-seen concealed objects.

With our investigation of the LVC framework, adapted for concealed object detection in (sub-)THz images, we observed a significant (RGB to sub-THz) domain gap that affects the verification efficiency when using the ViT. This discrepancy arises because the ViT was originally developed to be trained on natural RGB images. As a result, with LVC, the similarity scores between initial candidates and various categories of ground truth objects are closely matched, posing a challenge in verifying the labels of pseudo-annotations. This issue ultimately leads to the degraded performance of object detectors in the sub-THz domain, when compared to the RGB domain. To address this issue, we have modified the verification process of pseudo labels and introduced our improved pseudo-annotation (IPA) method to source high-quality candidates from unlabeled sub-THz image datasets. By employing multiple detectors, trained for a single concealed object category, we can localize initial candidate objects in the unlabeled images; even those with relatively low probability scores. Subsequently, a fine-grained classifier is applied to identify the ‘difficult to distinguish’ objects and provide new classification scores. This classifier is pre-trained on the supporting thermal-infrared image dataset and then fine-tuning on the open-source sub-THz image dataset.

Inspired by another thermal-infrared application^23–25^, down-sampling thermal-infrared images share similar spatial features to (sub-)THz images that can be used for regularization^26^ (i.e., preventing the classifier from overfitting) during the pre-training process. After obtaining these high-quality pseudo-annotations, we fine tune the object detector that is trained using the TFA framework. With our proposed method, the mean average precision (mAP) of Faster-RCNN in detecting ‘novel classes’ objects has significantly improved by almost 30%, from 22.8 to 29.5 (given a relatively small value for the few-shot number, *K* = 10, which represents the number of images required for training the detector), while still maintaining high performance in detecting ‘base classes’ objects.

## Related works

### Faster-RCNN

**Figure 1:**
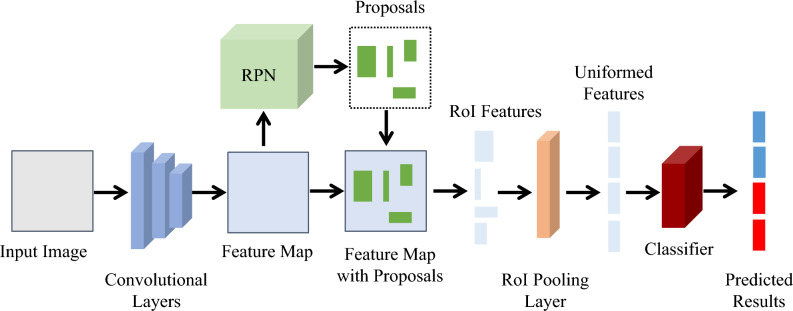
Architecture of the Faster-RCNN.

Faster-RCNN, introduced by Ren et al*.*^3^, is considered one of the leading solutions for object detection in visual imaging applications. It has also been widely adopted and enhanced for detecting objects in the (sub-)THz domain^10–12^. In the two-stage framework of Faster R-CNN, as illustrated in Fig. 1, objects are detected and classified by using three critical components: (1) convolutional layers; (2) regional proposal network (RPN); and (3) regions of interest (RoI) pooling layer, followed by a classifier. By calculating the dot products between learnable kernel filters and an image, the convolutional layers extract a feature map that represents the image's convolutional features. This feature map is then utilized by the RPN, to predict the bounding boxes of objects and the ‘objectness’ scores at each position. During the training process, the RPN generates nine types of anchor boxes at each point on the feature map, using three scales and three aspect ratios. Since each anchor box contains either objects or background elements, the region proposals of the RPN are derived by learning to classify anchor boxes as positive or negative. Concurrently, the RPN predicts the offset and scale difference between the anchor boxes and the ground truths. Subsequently, the RoI features (cropped from these region proposals on the feature map) are transformed into a uniform dimension by the RoI pooling layer. This fixed-size RoI feature enables the classifier to learn to predict the location and categories, thereby achieving the function of localizing and recognizing the target objects in an image.

### Fine-tuning based frameworks

**Figure 2:**
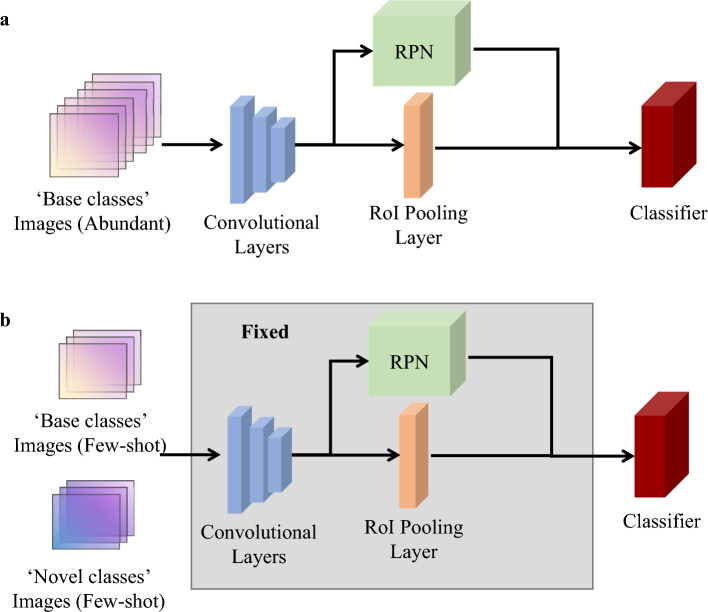
Overview of the TFA method stages: (**a**) base training; (**b**) fine-tuning.

In the application scenario of FSOD, Wang et al*.*^15^ discovered that by merely fine-tuning the last layer of Faster-RCNN, using balanced data from both ‘base classes’ and ‘novel classes’ objects; the detection performance for the latter objects can be markedly improved without compromising the performance for the former objects. This 2020 discovery led to the development of the Two-stage Fine-tuning Approach (TFA), illustrated in Fig. 2. The TFA framework consists of two principal training stages: (1) ‘base training’ stage; and (2) ‘fine-tuning’ stage. With the former, the entire Faster-RCNN is trained solely on a dataset containing ‘base classes’ objects. Transitioning to the latter, the components of the convolutional layers, RPN and RoI pooling layers remain fixed; only the classifier is fine-tuned using the balanced dataset containing objects from both few-shot ‘based classes’ and ‘novel classes’ categories. Due to its straightforward strategy and exceptional performance across various benchmarks for RGB image datasets, TFA has emerged as one of the leading solutions for FSOD applications.

**Figure 3:**
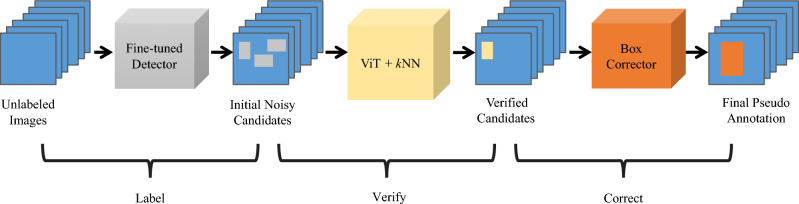
Overview of the LVC method.

Kaul et al*.*^16^ highlighted two main limitations associated with the TFA approach: ‘supervision collapse’ phenomenon^27^ and class imbalance among the categories within the dataset. The former is where a model discards information not directly related to its training class. For example, during the ‘base training’ stage, TFA overlooks the presence of ‘novel classes’ objects, thus, degrading the performance of an object detector. To address the challenges of ‘supervision collapse’ and class imbalance, the LVC framework is introduced by Kaul et al*.*^16^. This machine learning framework aims to generate high-quality pseudo-annotations from extra unlabeled images, for re-training the object detector, as illustrated in Fig. 3. Initially, the noisy candidates are extracted from unlabeled images using a detector that is trained with the TFA framework. The feature vectors of these noisy candidates are then extracted by a ViT^17^, which is trained using the self-supervised DINO method^28^. Cosine similarities between the feature vectors of the noisy candidates and the ground truth objects are calculated. Consequently, the top *k*-nearest neighbors (*k*NN) of the ground truth objects form a committee to vote on the class labels for these noisy candidates. Following this, a specialized box corrector, trained to predict the scale and offset difference between the proposals in the Faster-RCNN and ground truth, refines the size and locations of bounding boxes for these pseudo-annotations.

## Methods

### Sub-THz and thermal infrared cameras

The initial open-source dataset originates from the China Academy of Engineering Physics and is captured using an experimental active sub-THz imaging system, operating at 140 GHz in a scanning array mode, with a 5 mm × 5 mm scanning resolution and 335 $\times$ 880 pixel resolution^29^. The dataset from Imperial College London is used for pre-training the fine-grained classifier, employing a commercial thermal infrared FLIR E60 camera. This passive camera is an uncooled micro-bolometric focal plane array, having a spectral range from 7.5 to 13 µm (i.e., operating over a 17 THz thermal-infrared noise bandwidth, from 23 to 40 THz) with a 320 $\times$ 240 pixel resolution^30^.

### Improved pseudo-annotation

**Figure 4:**
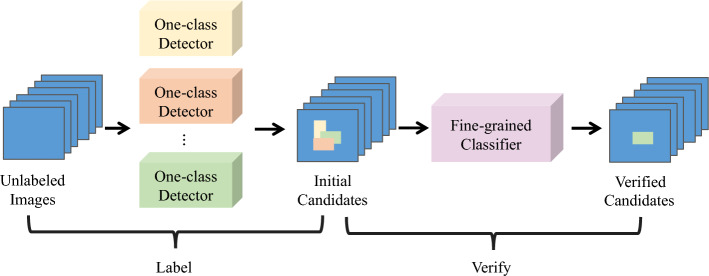
Overview of IPA method.

Within the LVC framework, the ViT was originally trained on natural RGB images. This creates a domain gap problem when applying the LVC framework to (sub-)THz images for FSOD applications. During the verification stage for LVC, the similarity scores between initial candidates and various categories of ground truth objects are closely aligned. To address this issue, we introduce the IPA method, as illustrated in Fig. 4. Our IPA machine learning framework, much like the workflow for the LVC method, consists of two main stages: (1) labeling stage; and (2) classification stage. Notably, there is no requirement for correcting the bounding boxes for the initial candidates, because both the localization and dimensions of the bounding boxes from the labeling stage are consistently accurate.

In contrast to the labeling stage of the LVC framework, we apply multiple one-class detectors instead of a fine-tuned detector. These one-class detectors are trained on the subsets of the sub-THz images containing a single category of concealed object. By using these detectors, the initial candidates are annotated with the coordinates of the bounding boxes and a confidence score that is specific to each class of concealed object. Employing multiple one-class detectors helps to mitigate the class-imbalance problems within the training dataset.

During the classification stage, we replace the ViT model with a fine-grained classifier, which is designed to re-verify the ‘novel classes’ objects with updated class labels and confidence scores. This adjustment offers a more reliable and flexible label verification of initial candidates, particularly for concealed objects like ceramic knives and metal dagger, having similar appearance. Given that certain classes of objects have either sufficient numbers of samples or distinct features, the fine-grained classifier can target specific subsets for the initial candidates. With our implementation, the classifier is utilized to re-classify the initial candidates with the following label classes: ‘scissors’, ‘gun’, ‘metal dagger’ and ‘ceramic knife’.

**Figure 5:**
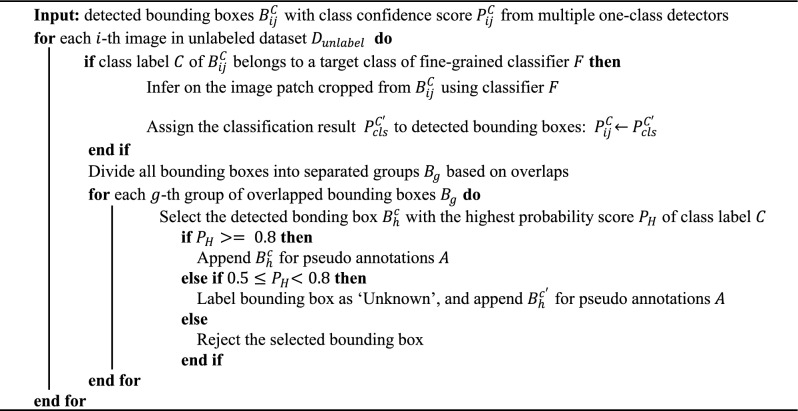
Algorithm for implementing the IPA method. For the *i*th image in the unlabeled dataset, multiple one-class detectors produce the *j*-th candidate bounding box ${B}_{ij}^{C}$ specific to class *C*. If the class label $C$ belongs to a target class of the fine-grained classifier $F$, then classifier $F$ is applied to the image patch cropped from ${B}_{ij}^{C}$ and the new class confidence score will be assigned based on the classification result. Within the same image, candidates with overlapping bounding boxes are grouped together and the candidate with the highest confidence score is selected. We use the empirical value of 0.8 as a threshold for accepting candidates, which aligns with the LVC framework proposed by the Oxford VGG group. Candidates with confidence scores below 0.5 are rejected using a common default threshold value. Within the open-source THz image dataset, there are items labeled as ‘Unknown’ that exhibit inconsistent appearances. Consequently, we have designated objects with confidence scores ranging from 0.5 to 0.8 as ‘Unknown’.

Regarding the model setting, each one-class detector uses the Faster-RCNN architecture, with ResNet-50^31^ as its convolutional layers. The fine-grained classifier employs ResNet-18 for feature extraction. By using an early stopping strategy, this classifier is pre-trained on the higher resolution thermal-infrared images that is downscaled. This approach is supported by previously published research^26^, which suggests that it can enhance the model’s robustness and uncertainty, especially when the fine-tuning dataset is limited in size. Subsequently, the classifier is fine-tuned on the image patches that have been cropped from the bounding boxes of the ground truth objects in the training sub-THz image dataset. Apart from the target categories of the concealed objects for the fine-grained classifier, other classes of initial candidates are filtered, based on the confidence score of their bounding boxes. After collecting all the initial candidates, we perform a selection of pseudo-annotations for final candidates, as summarized in the following pseudo code given in Fig. 5.

## Experiments and results

### Sub-THz imaging dataset and few-shot experiments

We utilized the open-source low-contrast active sub-THz image dataset^29^ to develop and evaluate the efficacy of Faster-RCNN, using different FSOD machine learning frameworks. The dataset contains 3157 images (each having 335 $\times$ 880 pixels) in total, with 11 classes of concealed objects. Each image in the dataset displays human models with up to three concealed objects under their clothing, with examples shown in Supplementary Fig. S1 online.

To emulate real-world conditions of FSOD, frequently seen items (e.g., water bottles, leather wallets, cigarette lighters, key chains, and cell phones) are considered as ‘base classes’ objects. In contrast, less common but potentially dangerous items (e.g., scissors, guns, metal daggers, ceramic knives, Chinese kitchen knives, and unknown items) are designated as ‘novel classes’ objects. For a balanced evaluation, the dataset is partitioned into a 60:40 ratio for corresponding training and testing purposes. The training dataset only samples *K*-shot instances (with $K=1, 3, 5, 10$) of ‘novel classes’ object images. The remaining images, including those with mixed ‘base classes’ and ‘novel classes’ objects, were treated as an unlabeled dataset by discarding their annotations. The test dataset is a ‘holdout’ dataset (i.e., not used for training) that contains all mask information (ground truth) for evaluating the algorithm.

The experimental environment is Ubuntu 20.04, CPU model 11th Generation Intel (R) Core (TM) i9-11, 900 K @3.50 GHz, GPU model NVIDIA GeForce RTX 3090, memory 24 GB. All detectors are trained using the stochastic gradient descent (SGD) algorithm with a mini-batch size of 16, momentum of 0.9, and weight decay of 0.0001. The initial learning rate of 0.02 is used during base training stage and 0.001 during fine-tuning stage.

### Dataset for pre-training the fine-grained classifier

**Figure 6:**
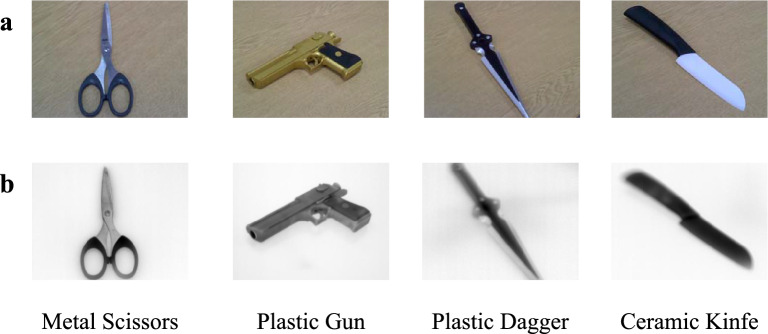
Examples of object images using our commercial FLIR E60 camera: (**a**) visible domain (RGB images); (**b**) thermal-infrared domain for pre-training the classifier.

The 200 thermal-infrared images were divided into four categories, with 50 separate images of the following: pair of metal scissor, all plastic toy gun, all plastic toy dagger, and a ceramic kitchen knife. These common object images were taken from various angles and positions, with examples shown in Fig. 6. Data collection is conducted at room temperature, however, to ensure a good contrast between the objects and background, the items were refrigerated to approximately $4^\circ{\rm C}$ prior to image capture.

### Evaluation metrics

Average precision (AP) is the metric employed to measure the accuracy of object detectors. The value of AP can be calculated^32^ using Eq. (1):

1 $$AP = {\sum }_{n=0}^{N}\left(R\left(n\right)- R\left(n+1\right)\right){Pr}_{interp}(R\left(n\right))$$

where $n$ represents each individual prediction made by the model. $R(n)$ is a set of reference recall values, sorted by confidence score (from high to low). ${Pr}_{interp}$ is the interpolated precision value at each recall level. The Eq. (1) approximation is normally represented by a percentage for the normalized area under the precision-recall curve.

The values of precision $Pr$ and recall $R$ can be computed using Eqs. (2) and (3):

2 $$Pr = \frac{TP}{TP+FP}$$

3 $$R = \frac{TP}{TP+FN}$$

A true positive (TP) arises when the label of positive detection matches the actual class; a false positive (FP) occurs when the label of positive detection is incorrect or mistaking the background as an object; and a false negative (FN) appears when the actual object is undetected. For the object detection task, the positive detection is defined by a specific Intersection-over-Union (IoU) threshold value. The IoU is the ratio of the intersection area between the detected and ground truth bounding boxes to the union area of these two boxes. The IoU threshold is commonly set at 50%; hence, the AP is often referred to as AP50. Within the context of few-shot object detection, the AP values associated with ‘base classes’ and ‘novel classes’ objects are specifically referred to as bAP and nAP, respectively. In this paper, we employ nAP50, bAP50, and mAP50 to evaluate the detection performance of object detectors with respective ‘novel classes’, ‘base classes’, and mean average precision of all objects.

### Comparisons with fine-tuning methods

**Figure 7:**
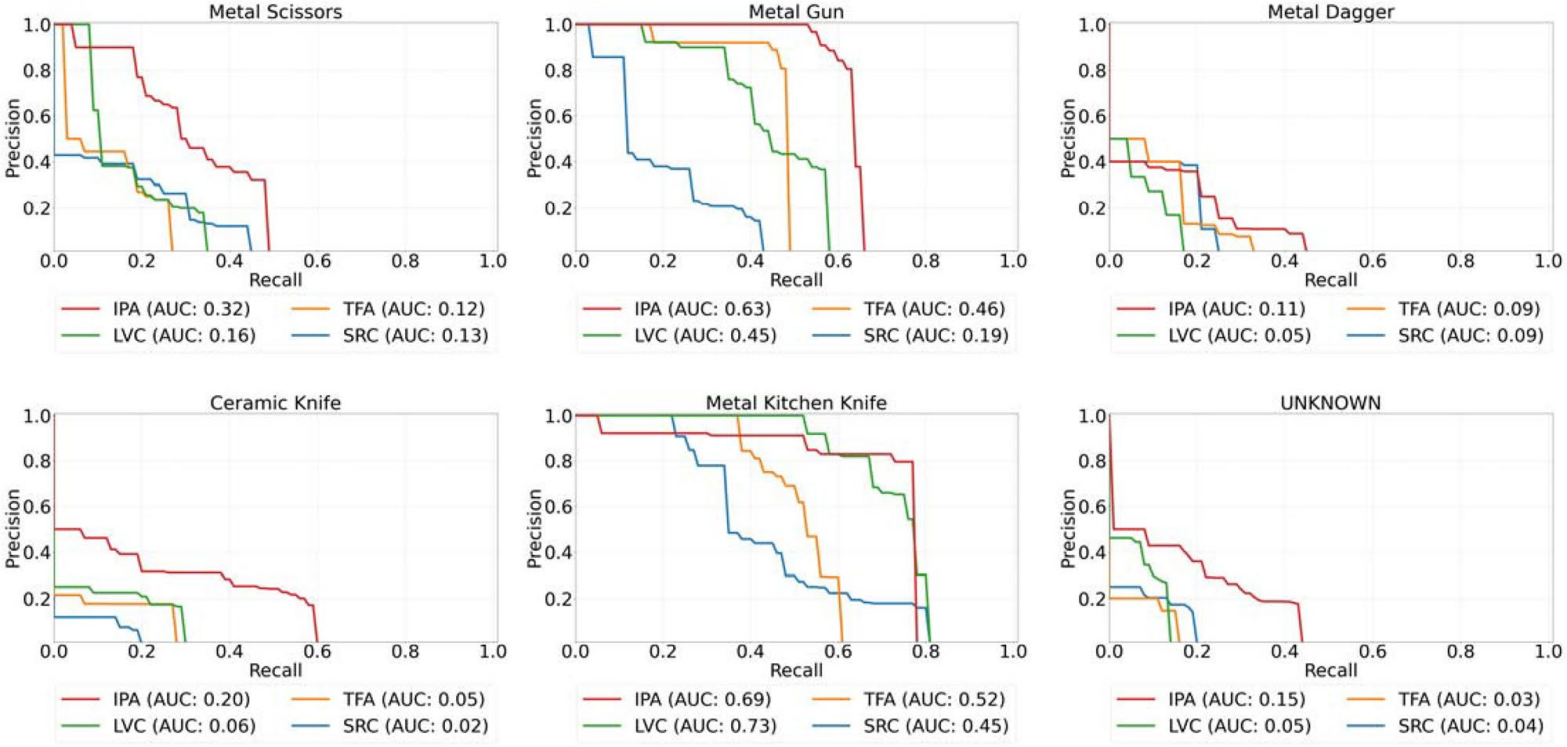
Precision-recall curve for $K = 10$ on ‘novel classes’ objects in the sub-THz test dataset. AUC represents the ‘area under the curve’ for various training methods. The ‘Unknown’ class refers to the unknown category items from the original open-source sub-THz image dataset having inconsistent appearances; being distinct from the defined concealed object categories.

To complement the experiment, the Faster-RCNN was also trained on the few-shot sub-THz image dataset directly, referred to as ‘source-only’ (SRC). The performance of the object detector using training strategies: SRC, TFA, LVC, and IPA are evaluated. According to the results shown in Table 1, the IPA method enhances the detector's capability in identifying ‘novel-classes’ objects, as reflected by the highest nAP values. It also sustains strong performance in detecting ‘base-classes’ objects, as evidenced by the relatively high bAP values. This trend is consistent across most few-shot settings, except for cases where the number of shots $K = 1$. In terms of detecting each category of ‘novel classes’ objects, with the exception of the metal kitchen knife, the IPA method outperforms all other machine learning frameworks; displaying a larger area under the precision-recall curve, as shown in Fig. 7.

**Table 1:** Experimental results for the Faster-RCNN detector using different FSOD training strategies.

Methods	Average precision (%) for $K=1, 3, 5, 10$											
	nAP50				bAP50				mAP50			
	1	3	5	10	1	3	5	10	1	3	5	10
SRC	1.5	5.8	9.6	12.3	35.3	*38.0*	41.0	44.3	14.8	17.6	21.0	25.2
TFA	**4.8**	*7.8*	12.6	14.6	**41.8**	37.9	**47.7**	**48.5**	**21.6**	*21.5*	**30.7**	*30.5*
LVC	*2.0*	4.9	*17.5*	*22.8*	25.3	24.4	36.8	37.2	12.6	13.7	26.3	28.7
IPA (our results)	1.2	**12.7**	**20.9**	**29.5**	*41.7*	**43.5**	*45.2*	*47.4*	*20.9*	**25.1**	*29.9*	**36.1**

Best and second-best results are colored bold and italic, respectively.

Due to the RGB to sub-THz domain gap issue, which undermines the verification process, the LVC framework did not exhibit superior performance in detecting ‘novel-classes’ and ‘base-classes’ objects amongst all the few-shot settings, even with additional pseudo-annotations from unlabeled images. This can be attributed to the fact that the self-supervised ViT model, utilized within the LVC, was initially trained on RGB images, which is not adapted for object detection in sub-THz images.

### Efficacy of using improved pseudo-annotations

**Figure 8:**
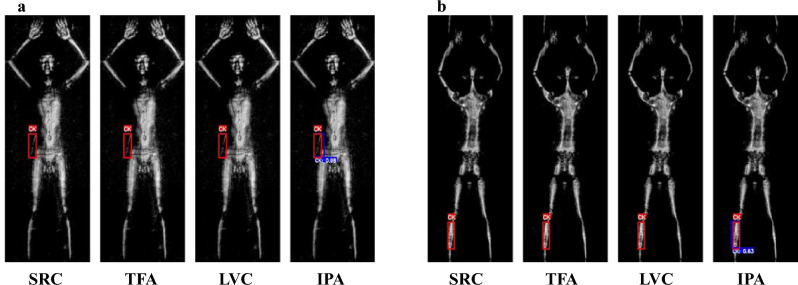
Examples of detecting ceramic knives from a test dataset (few-shot $K=10$). (**a**) Detection of a ceramic knife in an image with person facing forwards. (**b**) Detection of a ceramic knife in an image with person facing backwards. For each row of four images, from left to right, the detection results are for the: SRC, TFA, LVC, and IPA methods. The bounding boxes in red represent the ground truth, given by the original dataset, and in blue our detection result. The white text ‘CK’ represents the abbreviation for ‘Ceramic Knife’ and the associate number corresponds to the confidence score. The confidence score and coordinates of the bounding box (in blue) are obtained directly from the output of the object detection model, trained from FSOD frameworks.

Using pseudo-annotations from unlabeled images can augment the training dataset, even for detecting challenging object such as ceramic knives. By employing multiple one-class detectors, the candidates of ‘novel classes’ objects from unlabeled images with lower probability scores are detected. When a fine-grained classifier is applied, the candidates are verified as pseudo-annotations and used for further training the object detector; enhancing the performance of the object detector, as illustrated in Fig. 8. Furthermore, examples of enhancement in detecting both ‘base classes’ and ‘novel classes’ objects using our IPA method can be found in Supplementary Figure S2 online.

However, during the sourcing of candidates from the unlabeled dataset, a threshold value of 0.5 is used to exclude candidates with lower confidence scores; and objects with confidence scores ranging from 0.5 to 0.8 are categorized as ‘Unknown’. Instances exhibiting inconsistent features that do not match those in the training dataset are more likely to receive the ‘Unknown’ label. This inconsistency leads to lower confidence scores and increases the potential for pseudo annotations to be labeled as ‘Unknown’.

## Result discussion

The main limitation of our method is that the quality of the pseudo-annotations is still dependent on the training dataset. When training the fine-grained classifier and the Faster R-CNN within our FSOD framework, the performance of these machine learning models is positively correlated with the number of few-shot *K* ‘novel classes’ images. This is due to the data-driven nature of the CNN utilized by these models. When the few-shot number $K=1$, our IPA method performs poorly, due to the limited presence of ‘novel classes’ objects for training a robust classifier. Training on cross-domain (sub-THz and thermal-infrared) images can introduce beneficial regularization effects to prevent overfitting, compared to simply training a classifier on a limited dataset. It is anticipated that further enhancements to our method can be made if additional close-support domain images are available (e.g., images collected from another (sub-)THz imaging device). In addition, the potential advanced deep learning techniques, such as domain adaptation (DA), may be able to assist in transferring the capabilities of machine learning models from the visual domain to the (sub-)THz domain.

### Supplementary Information

Supplementary Information.

## Data Availability

The sub-terahertz image dataset can be accessed at: https://github.com/LingLIx/THz_Dataset. The thermal-infrared image dataset for training the fine-grained classifier is available from the corresponding author upon reasonable requests.
