# Supplementary material for: Few-shot concealed object detection in sub-THz security images using improved pseudo-annotations
Source: Sci Rep. 2024 Feb 7;14:3150. doi: 10.1038/s41598-024-53045-9 (PMC10850053; doi:10.1038/s41598-024-53045-9)
Supplement: Supplementary file 1 — Supplementary Information. [file 41598_2024_53045_MOESM1_ESM.pdf]

S1. Examples of annotated Sub-THz images

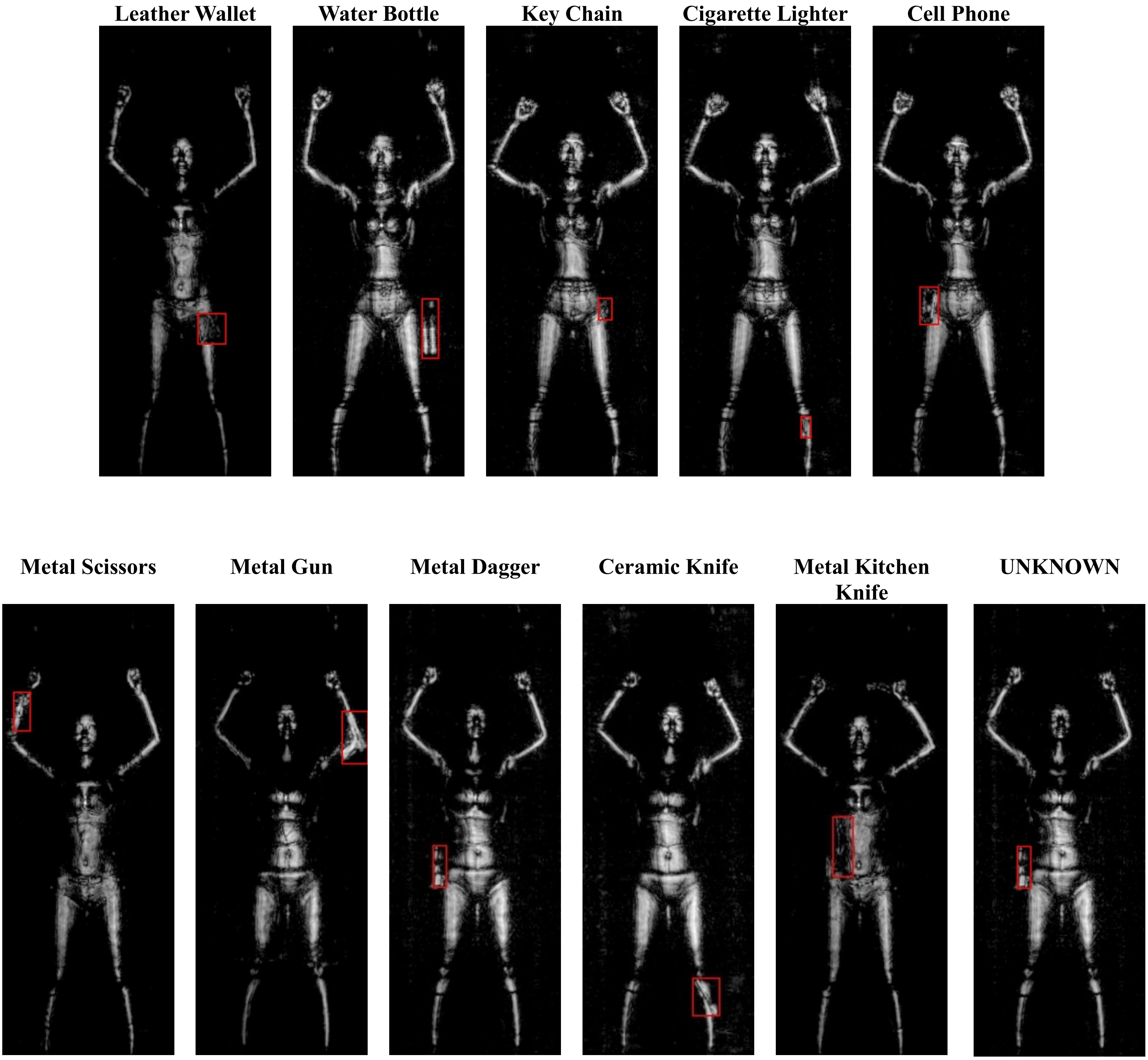

(a) Examples of annotated sub-THz images with a person facing forwards: the top 5 categories are 'base classes' objects, and the bottom 6 categories are 'novel classes' objects.

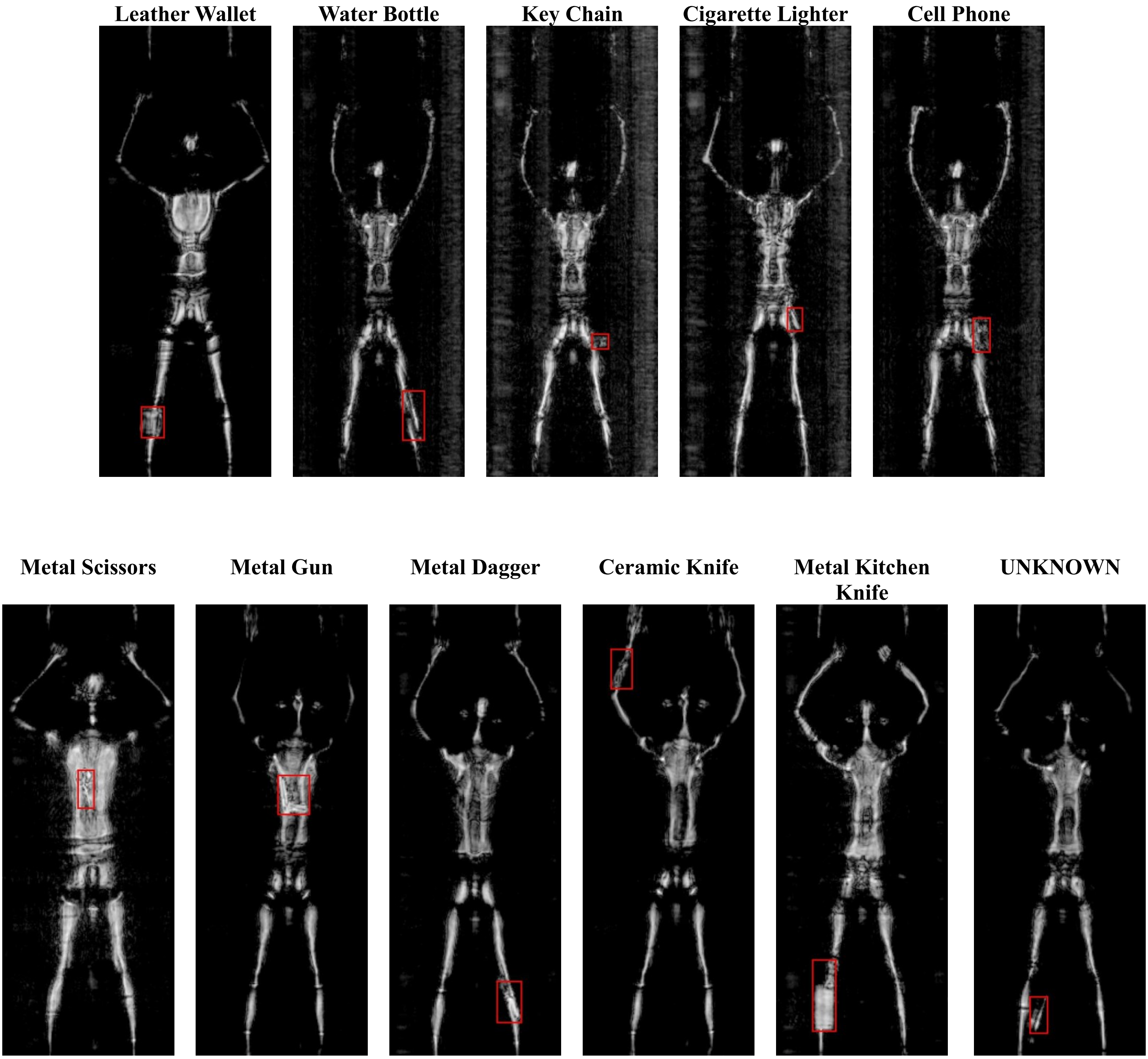

(b) Examples of annotated sub-THz images with a person facing backwards: the top 5 categories are 'base classes' objects, and the bottom 6 categories are 'novel classes' objects.

S2. Examples of detection results from a test dataset (few-shot $K = 10$) by utilizing various FSOD frameworks

For each row of four images, from left to right, the detection results are for the: SRC, TFA, LVC, and IPA methods. The bounding box in red represents the ground truth, and in blue the detection result. The white text represents the abbreviation for object categories and the associate number corresponds to the confidence score. (a)-(c) are detection results for the objects of interest: metal gun, metal dagger and metal scissors where the fine-grained classifier applied in our IPA framework. The remaining images contain either 'base classes' objects, 'novel classes' objects, or both.

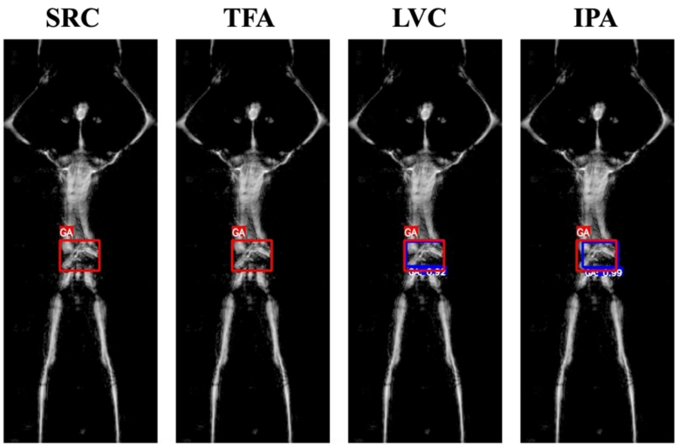

(a) Detection of a **metal gun** in an image with the person facing backwards.

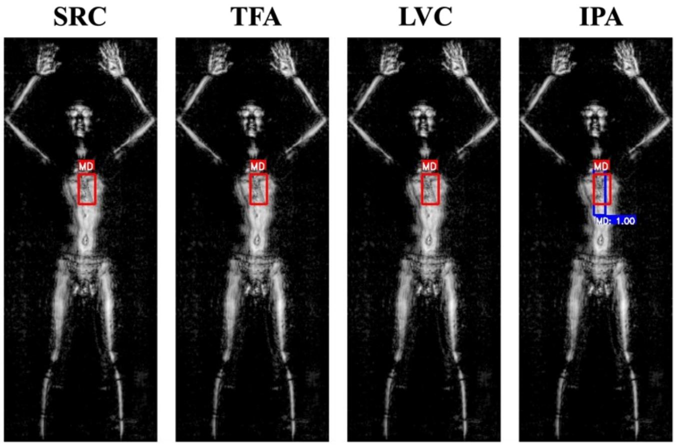

(b) Detection of a **metal dagger** in an image with the person facing forwards.

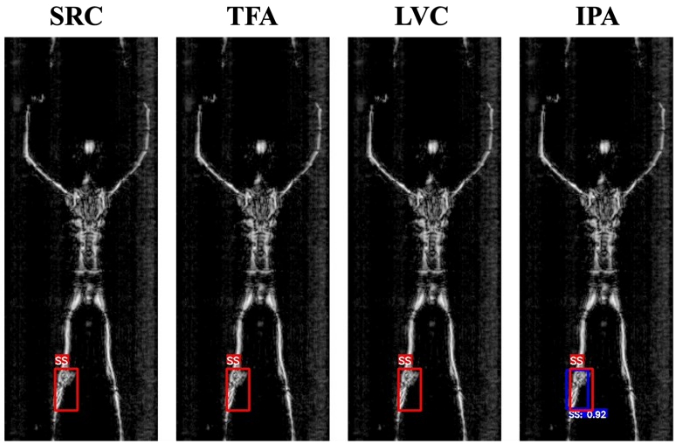

(c) Detection of a **metal scissors** in an image with the person facing backwards.

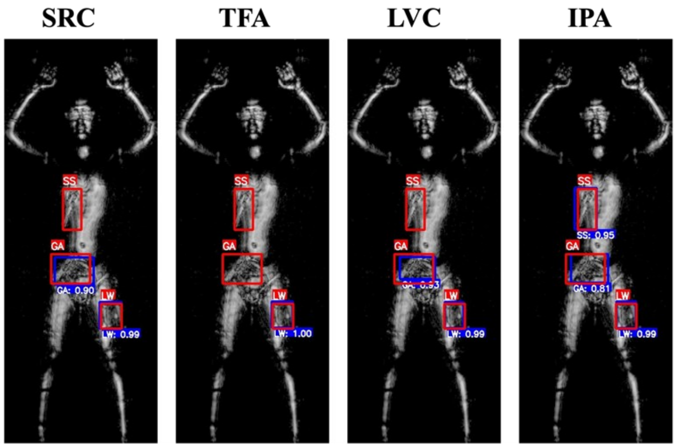

(d) Detection of one **metal gun**, one **leather wallet,** and a pair of **metal scissors** in an image with the person facing backwards.

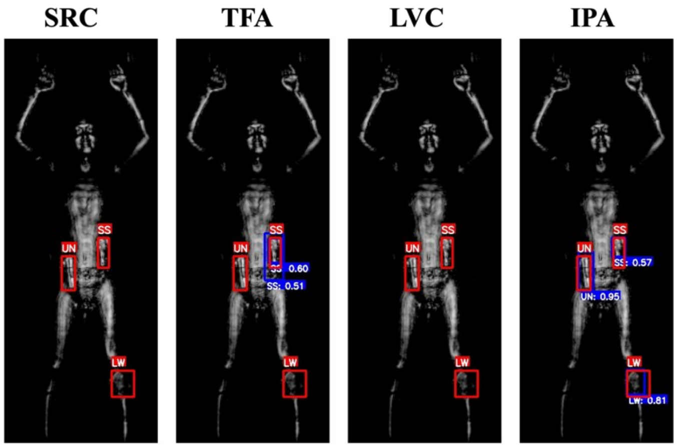

(e) Detection of one **UNKNOWN** object, one pair of **metal scissors**, and one **leather wallet** in an image with the person facing forwards.

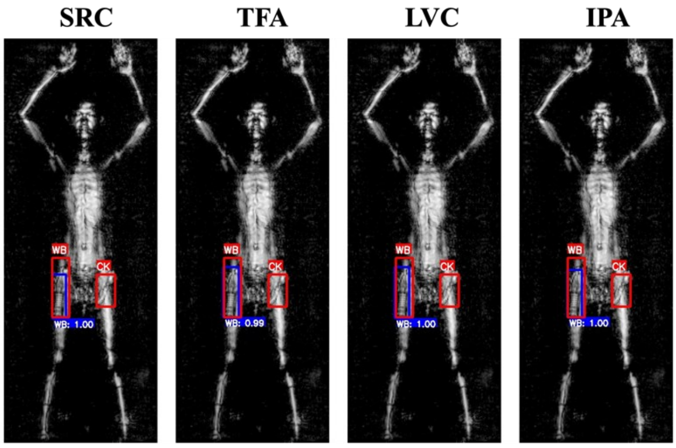

(f) Detection of one **water bottle** and one **ceramic knife** in an image with the person facing forwards.

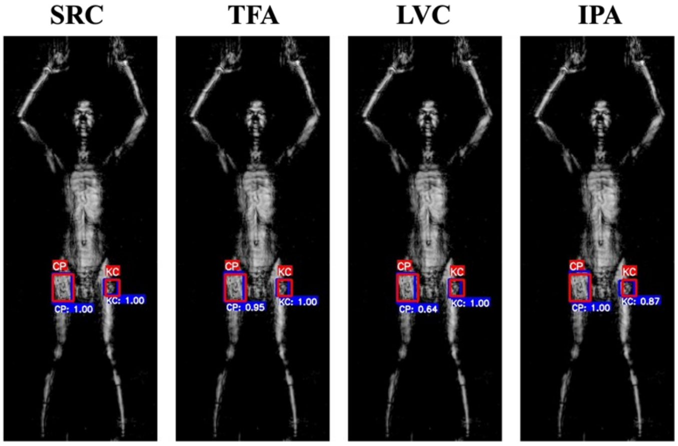

(g) Detection of one **cell phone** and one **key chain** in an image with the person facing forwards.

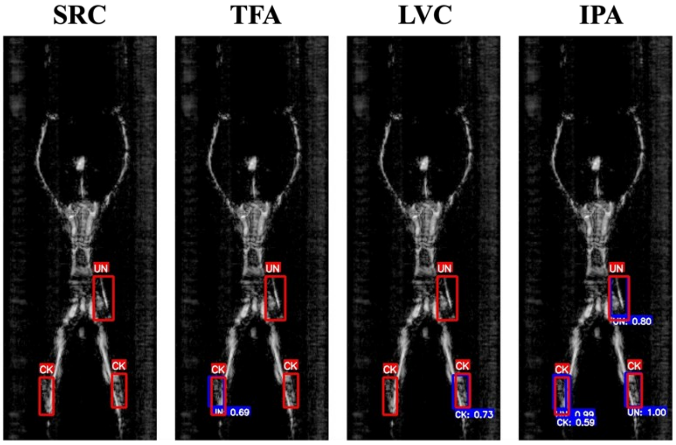

(h) Detection of one **UNKNOWN** object and two **ceramic knives** in an image with the person facing backwards.

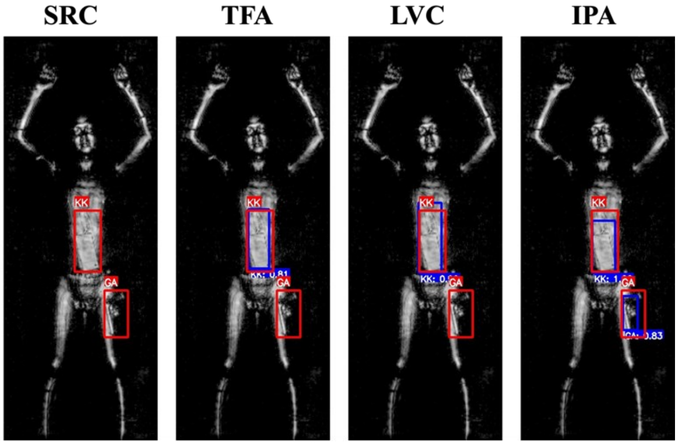

(i) Detection of one **kitchen knife** and one **metal gun** in an image with the person facing forwards.
